# Analysis of the HIV resistance related HLA dRB1 restricted CD4+ T cell epitopes among Indian HIV patients

**DOI:** 10.1186/1471-2334-14-S3-O4

**Published:** 2014-05-27

**Authors:** H Jemmy Christy, D Alex Anand

**Affiliations:** 1 Department of Bioinformatics, Sathyabama University, Chennai-600119, India

## Background

Host genetic factors are the major determining factor for variation in response to HIV infection and are related to resistance among HIV patients. HLA dRB1*1501, DRB1*1502, DRB1*1301, DRB1*1302, DRB1*1101-05 are widely reported alleles associated with low viraemia in infected individuals. Computational predictions have made it possible to identify potentially strong peptide binders to HLA molecules that so that can be then tested *in vitro* and *in vivo* as putative epitopes for peptide based vaccines. The present study investigated the consequences of sequence variations within HLA dRB1 alleles in selecting the epitopes and their interactions.

## Methods

V3 loop based epitope prediction with their binding values restricted to HLA dRB1 alleles were generated using the IEDB, ANN prediction server and Propred II, a Quantitative matrix based method. Population coverage was calculated for selected DRB1 alleles by the Population coverage tool on the IEDB server. We modeled consensus v3 loop based epitopes Pepstr a peptide modeling tool and HLA dRB1*1501, DRB1*1502, DRB1*1301, DRB1*1302, DRB1*1101-05 using I tasser, an ab initio modeller, interaction studied between HLA alleles and epitopes using docking simulation models generated by Flexpepdock.

## Results

Out of 189 predicted low percentile ranked epitope, TRKSIRIGPGQTFYA, NNTRKSIRIGPGQTF, YATGDIIGDIRQAHC, NTRKSIRIGPGQTFY were observed with consensus and were found interacting strongly with the HIV resistance related HLA alleles and visualized in Discovery studio visualize 3.5.

## Conclusion

The epitope specific to HLA dRB1 alleles have been identified by different methods indicating their potential role as candidate epitope for an effective HIV vaccine.

